# Identification of Modulators of the *C.* *elegans* Aryl Hydrocarbon Receptor and Characterization of Transcriptomic and Metabolic AhR-1 Profiles

**DOI:** 10.3390/antiox11051030

**Published:** 2022-05-23

**Authors:** Lucie Larigot, Linh-Chi Bui, Marine de Bouvier, Ophélie Pierre, Grégory Pinon, Justine Fiocca, Mohammad Ozeir, Cendrine Tourette, Chris Ottolenghi, Sandrine Imbeaud, Clément Pontoizeau, Benjamin J. Blaise, Aline Chevallier, Céline Tomkiewicz, Béatrice Legrand, Bénédicte Elena-Herrmann, Christian Néri, Vanessa Brinkmann, Pierre Nioche, Robert Barouki, Natascia Ventura, Julien Dairou, Xavier Coumoul

**Affiliations:** 1 INSERM UMR-S1124, T3S, Toxicologie Environnementale, Cibles Thérapeutiques, Signalisation Cellulaire et Biomarqueurs, Université Paris Cité, 75006 Paris, France; luciole271@gmail.com (L.L.); thanhntc@yahoo.com (L.-C.B.); marinedebvr@gmail.com (M.d.B.); ophpierros@gmail.com (O.P.); gregory.pinon@inserm.fr (G.P.); fiocca.justine@gmail.com (J.F.); mohammad.ozeir@hotmail.fr (M.O.); chris.ottolenghi@parisdescartes.fr (C.O.); aline.chevallier@gmail.com (A.C.); celine.tomkiewicz@inserm.fr (C.T.); beatrice.le-grand@parisdescartes.fr (B.L.); pierre.nioche@gmail.com (P.N.); robert.barouki@parisdescartes.fr (R.B.); 2 CNRS UMR 8601, Metabolism, Pharmacochemistry and Neurochemistry, Université Paris Cité, 75006 Paris, France; 3 Unité de biologie fonctionnelle et adaptative, UMR 8251, CNRS, Université Paris Cité, 75013 Paris, France; 4 Laboratoire Interactions Epithéliums-Neurones (LIEN), Université de Brest, EA4685, 29200 Brest, France; 5 Structural and Molecular Analysis Platform, Biomedtech Facilities, Université Paris Cité, 75006 Paris, France; 6 Centre Paul Broca, INSERM U894 Neuronal Cell Biology & Pathology & EA Université Paris Cité, 75014 Paris, France; cendrinetourette@gmail.com; 7 AP-HP, Hôpital Necker-Enfants Malades, Service de Biochimie Métabolique, 75015 Paris, France; clement.pontoizeau@gmail.com; 8 Gif/Orsay DNA MicroArray Platform, 91190 Gif sur Yvette, France; sandrine.imbeaud@crc.jussieu.fr; 9 Centre de Résonance Magnétique Nucléaire à Très Hauts Champs, Univ. Lyon, CNRS, UCBL, ENS Lyon, 69100 Villeurbanne, France; benblaise@gmail.com (B.J.B.); benedicte.elena@univ-grenoble-alpes.fr (B.E.-H.); 10 Institute for Advanced Biosciences, Univ. Grenoble Alpes, CNRS, INSERM, 38000 Grenoble, France; 11 CNRS UMR 8256, Inserm ERL U1164, Sorbonne Université, 75005 Paris, France; christian.neri@inserm.fr; 12 Institute of Clinical Chemistry and Laboratory Diagnostic, Medical Faculty, Heinrich Heine University, Düsseldorf, Moorenstr 5, 40225 Düsseldorf, Germany; vanessa.brinkmann@uni-duesseldorf.de (V.B.); natascia.ventura@uni-duesseldorf.de (N.V.); 13 Leibniz Institute for Environmental Medicine (IUF), Auf’m Hennekamp 50, 40225 Düsseldorf, Germany; 14 Assistance Publique-Hôpitaux de Paris, Hôpital Necker, 75015 Paris, France

**Keywords:** Aryl hydrocarbon Receptor, *Caenorhabditis elegans*, transcriptomics, metabolomics, modulators

## Abstract

The Aryl hydrocarbon Receptor (AhR) is a xenobiotic sensor in vertebrates, regulating the metabolism of its own ligands. However, no ligand has been identified to date for any AhR in invertebrates. In *C.* *elegans*, the AhR ortholog, AHR-1, displays physiological functions. Therefore, we compared the transcriptomic and metabolic profiles of worms expressing AHR-1 or not and investigated the putative panel of chemical AHR-1 modulators. The metabolomic profiling indicated a role for AHR-1 in amino acids, carbohydrates, and fatty acids metabolism. The transcriptional profiling in neurons expressing AHR-1, identified 95 down-regulated genes and 76 up-regulated genes associated with neuronal and metabolic functions in the nervous system. A gene reporter system allowed us to identify several AHR-1 modulators including bacterial, dietary, or environmental compounds. These results shed new light on the biological functions of AHR-1 in *C.* *elegans* and perspectives on the evolution of the AhR functions across species.

## 1. Introduction

The well-evolutionarily conserved Aryl hydrocarbon Receptor (AhR) is a transcription factor involved in the mediation of biological responses to environmental chemicals in vertebrates. After its discovery, its role in the regulation of the induction of drug-metabolizing enzymes and/or in the mediation of the toxicity of tetrachlorodibenzo-*p*-dioxin (TCDD) was an extensively studied function of the receptor [1,2,3]. This characterization of the AhR as a xenobiotic sensor was followed by the finding of many types of exogenous ligands including polycyclic aromatic hydrocarbons (PAHs), polyhalogenated hydrocarbons (dioxins), furans and polychlorinated biphenyls (PCBs) [4]. After binding to its ligands, the AhR translocates into the nucleus and heterodimerizes with its partner aryl hydrocarbon receptor translocator (ARNT). The active complex binds to xenobiotic responsive elements (XRE) localized in the 5′ region of targeted genes and modulates their expression after recruiting coregulators [5,6].

Today, additional functions of the transcription factor have been discovered; on the one hand through the identification of endogenous ligands such as tryptophan derivatives like 6-formylindolo[3,2-b]carbazole (FICZ) [7,8] and on the other hand because additional AhR physiological functions in vertebrates have been revealed by the characterization of the AhR Knockout (AhR KO) mice model, which displayed developmental anomalies, decreased fertility, vascular pathologies or immune impairment, described in addition to adaptive roles [9,10,11,12]. In addition, the implication of AhR was also highlighted in the development and homeostasis of the nervous system, and the regulation of neural functions, as described by the dysregulation of myelination or oculomotor deficits in AhR KO mice [13,14,15,16].

The evolutionary conservation of the AhR is another line of evidence that this transcription factor plays an important role in physiology. The best-described invertebrate AhRs are Spineless in *Drosophila melanogaster* (*D. melanogaster*) and AHR-1 in *Caenorhabditis elegans* (*C. elegans*). Orthologs found in invertebrate species indicate that the AhR has physiological functions, and initial studies showed that it did not appear to bind xenobiotics such as TCDD or β-naphthoflavone [17,18]. In invertebrates, the AhR orthologs are mostly expressed in neurons and most of the physiological roles described are neuronal functions. For example, *C. elegans* AHR-1 is involved in neuronal development as it specifies the cell fate of GABAergic [19] and sensory neurons [20] but also in cell migration and axonal guidance [21]. *D. melanogaster* Spineless plays a role in dendrite morphogenesis [22] or in the retinal mosaic that composed the eye unit [23]. So, the conclusion was that the AhR in invertebrates does not act as a sensor but rather as protein involved in neurodevelopment.

The inability of invertebrate AhRs to bind prototypical ligands of the vertebrate receptor suggests that the xenobiotic sensing function of the receptor in vertebrates is an adaptation acquired during evolution and is not its ancestral function. To challenge this conclusion and to better understand the physiological roles of AhR, one approach is to characterize its functions and regulations in other species, especially the ones in which no ligand of the receptor has been clearly characterized yet, such as *C. elegans*.

In this study, we followed two approaches to better understand the functions of AhR in *C. elegans* and to further understand the evolution of this protein functions. We first performed a metabolomic analysis and demonstrated that loss-of-function mutation in the *ahr-1* gene induces strong metabolic changes in *C. elegans* involving amino-acid metabolism, carbohydrate metabolism as well as fatty acid metabolism. The metabolic disruption of the endogenous functions of the AhR was previously suggested by several transcriptomic studies in *C. elegans* [24,25]. However, all these transcriptomic experiments were carried out on the whole nematode, while AHR-1 is mostly expressed in a small subset of neurons. Considering the expression profile of AHR-1 in *C. elegans*, we next examined the transcriptomic signatures in AHR-1-expressing neurons, comparing both wild-type (WT) and *ahr-1(ia03)* mutants, identifying several cytochromes P450 (CYP) as potential targets. As this novel observation could suggest a conservation of CYP regulation in evolution, we also developed a tool to screen potential modulators of AHR-1 using Cos-7 cells which do not express functional AhR. We identified positive and negative modulators of AHR-1 transcriptional activity and these results indicate the AHR-1 ability to be activated or repressed by environmental, dietary, and bacterial compounds. Considering the *C. elegans* environment, these results could help us to better understand the role of AhR in invertebrates and to provide insight into new features in AhR biology.

## 2. Materials and Methods

### 2.1. C. elegans Strains

N2 nematode strain was provided by the Caenorhabditis Genetics Center, which is funded by the National Institutes of Health National Center for Research Resources. *ahr-1(ia03)* mutant animals were obtained from the laboratory of J.A. Powell-Coffman Coffman (Department of Genetics, Development, and Cell Biology, Iowa State University, Ames, IA 50011-3260, USA). *ahr-1(ia03)* worms were crossed with wild-type animals carrying GFP marker in MEC3 touch neurons (*pmec3::gfp*) obtained from C. Neri’s lab, in order to obtain the homozygote hermaphrodite *ahr-1::gfp* mutant.

### 2.2. Primary Cultures of Embryonic Nematode Cells

Embryonic cells were obtained as previously described [26]. Embryos were isolated from gravid adults following lysis in a hypochlorite solution. Eggshells were removed by incubation in 0.5 mL chitinase/chymotrypsine (1 U/mL and 3000 U/mL, respectively, in egg buffer) for 20 min. Following resuspension in egg buffer, the embryos were dissociated by 0.25% trypsin treatment for 5 min and resuspended in L-15 supplemented with antibiotics and 20% FBS (L15-CM). Cells were plated on TESPA (4%, Sigma, Saint Quentin Fallavier, France) coated glass plates at a density of 300,000 cells/cm^2^ and maintained in L15-CM media. Cells were incubated at 20 °C overnight.

An enriched population of AHR-1 expressing neurons in both wild-type GFP and mutant AHR-1::GFP were generated using fluorescence activated cell sorting (FACS) in collaboration with Cendrine Tourette, Christian Neri (INSERM U894 Neuronal Cell Biology & Pathology & EA University of Paris 5, Centre Paul Broca, 2 ter rue d’Alesia 75014 Paris-France) and Anne Louise (Plateforme Cytométrie, Pasteur Institut, 25 rue du Docteur Roux, 75724 Paris cedex 15).

Cell sorting experiments were performed on a Dako Moflo flow cytometer (Agilent Technologies, Les Ulis, France). GFP-positive cells were directly collected in trizol LS (Invitrogen-Fisher Scientific, Ilkirch Graffenstaden, France) after sorting and total RNAs extracted according to the manufacturer’s protocol.

### 2.3. Microarray Experiments and Analysis

#### 2.3.1. Amplification, Labeling and Chip Hybridization

Four independent batches of total RNA from wild-type (N2 and WT-GFP) and mutant (*ahr-1(ia03)* and mutant-GFP) cells were isolated and the quality of the samples was analyzed using a bioanalyzer. Total RNA (4 “WT” samples; 4 “mutated” samples) was amplified with the MessageAmp kit (Invitrogen-Fisher Scientific, Ilkirch Graffenstaden, France). AhR-expressing neurons represent a small population of total nematode cells and huge quantities of *C. elegans* were cultured to allow the isolation of a significant quantity of RNA. For global transcriptomic analysis, an amplification step was thus critical to generating high quantities of mRNA from original small samples. After amplification, RNA concentration was determined using a nanodrop spectrophotometer then submitted to indirect Cy3/Cy5 labeling using the CyScribe Post Labeling Kit (GE Healthcare-Amersham Biosciences, France). Hybridization experiments were performed using the Agilent solutions and protocols including the *C. elegans* oligo microarray kit: more than 21,000 genes and transcripts are represented on each slide, which contains four identical 44 K microarrays (Godmap platform, CGM, Gif sur Yvette).

#### 2.3.2. Microarray Data Analysis

Array images were digitized using ScanArray (Agilent Technologies, Les Ulis, France) in XDR mode at 5 µm resolution and then quantified with Feature Extraction software (version 9.5.3). Genes differentially expressed between AHR-1 mutant and Wild type condition in the microarray study were identified by a multivariable permutation test controlling the false discovery rate (a moderated *t*-test with adjustment of *p*-values using the MAnGO software [27]). Genes were selected as significantly differentially expressed when the *p*-value was less than 0.05.

Microarray data were also examined for gene set enrichment analysis (GSEA, Broad Institute’s Molecular Signature Database). The microarray data can be accessed through the Gene Expression Omnibus accession no. GSE195728.

### 2.4. Quantitative Real-Time Polymerase Chain Reaction

Total RNAs were extracted using the RNeasy mini kit (Qiagen, Courtaboeuf, France) and reverse transcription was performed with each RNA sample using the WT-Ovation Pico RNA Amplification System (Nugen Technologies, Redwood City, CA, USA). Gene specific primers used for the real time PCR are available on Supplementary Table S4. Quantitative RT-PCR was carried out in a 10 μL reaction volume containing 40 ng of cDNA, 300 nM of each primer and ABsolute™ QPCR SYBR^®^ Green (Abgene-Courtaboeuf, France). Quantitative RT-PCR measurements were performed on an ABI Prism 7900 Sequence Detector system (Applied Biosystems-Fisher Scientific, Ilkirch Graffenstaden, France). PCR cycles consisted of the following steps: Taq activation (15 min, 95 °C), denaturation (15 s, 95 °C) and annealing and extension (1 min, 60 °C). The threshold cycle (Ct), which is inversely correlated to the amount of target mRNA, was measured as the number of cycles for which the reporter fluorescent emission first exceeds the background. The relative amounts of mRNA were estimated using the ΔΔCt method with *cdc42* (*C. elegans* RNAs) or *rpl13* (Cos-7 cells RNAs) as reference gene.

### 2.5. Whole C. elegans HR-MAS NMR Spectroscopy

#### 2.5.1. Sample Preparation

Worms were grown according to standard protocol except for a liquid step in worm amplification. Worms were fixed for 45 min in 1% paraformaldehyde and were then washed five times in distilled water followed by five washes in deuterium oxide. The assays were performed on large number of worms (40,000 worms for each condition, split into 1000 worms per analyzed NMR sample) prepared in at least three independent experiments. Additionally, 30 µL disposable Kel-f inserts with sealing caps for 4 mm NMR rotors were filled with around 1000 whole worms and frozen at −80 °C for storage until NMR analysis. Samples were thawed at room temperature 15 min before the NMR experiments.

#### 2.5.2. HR-MAS NMR Spectroscopy

*C. elegans* HR-MAS NMR spectroscopy was performed as previously described by Blaise et al. [28]. All experiments were carried out on a Bruker Avance II spectrometer, operating at 700 MHz (proton resonance frequency), equipped with a 4 mm HR-MAS probe. The temperature was controlled at 295 K throughout the experiments. For each sample, a 1D NOESY and a 1D CPMG 1H NMR spectrum was recorded to derive a metabolic profile. The magic-angle spinning frequency was set to 3.5 kHz. For each sample, a 1D NOESY 1H NMR spectrum was recorded to derive a metabolic profile. The spectral width was set to 12 ppm, and the acquisition time was 1.4 s, with a relaxation delay of 1.7 s for a total acquisition time of 13 min per spectrum (256 free induction decays co-added). The NOESY mixing time was set to 100 ms.

#### 2.5.3. HR-MAS Data Processing and Statistical Analysis

A 0.3 Hz exponential window functional was applied to the raw data prior to Fourier transform. NMR spectra were manually phased, and baseline corrected. The water area was excluded (d = 4.45–5.1 ppm) as well as DMSO and methanol signals (d = 2.71–2.75 ppm and d = 3.35–3.36 ppm). Data were normalized with the PQN process and mean-centered.

Unsupervised (Principal component analysis (PCA)) and supervised (Partial Least Square regression (PLS-DA) and Orthogonal Partial Least Square regression (OPLS-DA)) were performed. Univariate analysis was also carried out, using the non-parametric Mann–Whitney U test followed by the Benjamini–Hochberg correction for multiple testing.

### 2.6. Chemicals

Molecules tested were obtained from Sigma-Aldrich (Saint Quentin Fallavier, France) except for the 3-methylcholanthrene obtained from Supelco and TCDD obtained from Cambridge Isotope Laboratories. Tested substances were dissolved in DMSO, ethanol, nonane or water. Aliquots were stored in glass vials at –20 °C until further use except for hydrogen peroxide and TCDD which were prepared before each experiment.

### 2.7. Plasmid Construction

The Firefly luciferase reporter construct (p1A1-FL) was a gift from Pr. R. Barouki (UMRS 1124—Université Paris Cité). The plasmid is based on the pGL3-basic vector (Promega, Charbonnières les bains, France) containing the 5′ region of the human *cyp1A1* gene (−1566 to +73) upstream of the firefly luciferase coding sequence was described previously [29].

Renilla luciferase reporter vector pGL4.74 [hRluc/TK] was purchased from Promega (Charbonnières les bains, France).

The two vectors containing *ahr-1* and *aha-1* (pcDNA3-AhR-1-VP16 and pcDNA3-AhA-1-VP16) were a gift from G. Pinon (UMRS 1124—Université Paris Cité). The VP16 transcriptional activity domain was added after the two coding sequences of *ahr-1* and *aha-1* (BamHI site) and subcloned into the HindIII-ApaI double-digested pcDNA3 vector.

The *ahr-1(ju145)* mutant vector was obtained from pcDNA3-AhR-1-VP16 by site-directed mutagenesis (Stratagene QuickChange Site-Directed Mutagenesis Kit, Agilent Technologies, Les Ulis, France). The primer sequence 5′ to 3′ was: TGGTGTCTATGGACTAGAAAGTGTACGAGATGCTGG and the complement 5′ to 3′ was: CCAGCATCTCGTACACTTTCTAGTCCATAGACACCA (the bold nucleotide represents the C to T point mutation).

### 2.8. Cell Culture

Cos-7 cells (ATCC CRL-1651) were routinely cultured in Dulbecco’s Modified Eagle Medium, High Glucose, No glutamine, No Phenol Red (Gibco-Fisher Scientific, Ilkirch Graffenstaden, France) supplemented with 1 mM Sodium Pyruvate (Gibco-Fisher Scientific, Ilkirch Graffenstaden, France), Glutamax (Gibco-Fisher Scientific, Ilkirch Graffenstaden, France), Penicillin-Streptomicyn-Glutamin (Gibco-Fisher Scientific, Ilkirch Graffenstaden, France) and 10% Corning Foetal Bovine Serum (Fisher Scientific, Ilkirch Graffenstaden, France). The cells were cultivated in a CO_2_-enriched atmosphere (5%) at 37 °C.

### 2.9. Transfection and 48 Wells Plate Screening Protocol

Lipofectamine 2000 and the four plasmids (pcDNA3-AhR-1-VP16: 5 ng/well, pcDNA3-AhA-1-VP16: 5 ng/well, p1A1-FL: 244 ng/well and phRL-TK: 36 ng/well) were used for the transfection: Cos-7 cells were trypsinized and transfection was performed on cells in suspension in 10% FBS medium. Cells were plated at 20,000 cells/wells on Corning Biocoat Collagen 48 wells plates (Dutscher, Bernolsheim, France) and the medium was changed 4 h later, from 10% to 0% FBS. 24 h later, medium was changed, and treatments added in each well. Cells were lysed after 24 h of treatment with 65 µL of Passive Lysis Buffer (PLB) from Dual Luciferase Reporter Assay (Promega). Then, 20 µL of PLB/lysate per well were plated in a white 96-wells plate (Thermo Fisher Sterilin Microtiter-Fisher Scientific, Ilkirch Graffenstaden, France) and 100 µL of FireFly luciferin (LARII) were added before measuring the luminescence produced by the FireFly luciferase, then 100 µL of Stop & Glo were added before measuring the luminescence produced by the Renilla luciferase. Untreated Cos-7 cells were used as blank samples to measure the background signal. Luciferase activities were measured on a plate-reading luminometer (Enspire, PerkinElmer, Waltham, USA). Firefly luciferase assay was normalized to the Renilla luciferase to determine relative AHR-1 activity.

### 2.10. Cell Viability Assays

For each cell viability assay, cells were cultured, transfected and treated in the same conditions than described previously.

Neutral Red uptake assays were performed as described by Repetto et al., 2008 with Neutral Red powder from Sigma-Aldrich (Saint Quentin Fallavier, France). Briefly, after 24 h of treatment, Neutral Red uptake assay was performed by aspirate off medium from cells and add 200 µL of Neutral Red medium 0% FBS to each well. The plate was incubated for 2 h at 37 °C, then the Neutral Red medium was removed, and the cells washed with 150 µL PBS per well. Then, 300 µL of Neutral Red destain solution were added per well and the plate was rapidly shaken for 10 min. The OD of Neutral Red extract was measured at 540 nm using blanks which contain no cells as a reference.

Alamar Blue assays were carried out according to the manufacturer’s instructions (Fisher Scientific, Ilkirch Graffenstaden, France). The medium was removed, and the cells were rinsed with PBS. 200 µL of an Alamar Blue solution (10% Alamar Blue dye in DMEM 0% FBS) were added to the cells. In parallel, all molecules were tested on Alamar Blue without cells: each treatment was added on Alamar Blue solution and 200 µL were added to a 48 wells plate. The plates were incubated 4 h at 37 °C. The percentage reduction of Alamar Blue was calculated using absorbance readings at 570 nm and 600 nm.

$$\%\;\mathrm{reduction}\;\mathrm{of}\;\mathrm{Alamar}\;\mathrm{Blue}=\frac{(E_{oxy600}\times A_{600})-\left(E_{oxy570}\times A_{600}\right)}{\left(E_{red570}\times C_{600}\right)-\left(E_{red600}\times C_{570}\right)}\times 100.$$

*E_oxi_*_570_ = molar extinction coefficient (E) of oxidized alamarBlue Reagent at 570 nm = 80,586
*E_oxi_*_600_ = E of oxidized alamarBlue Reagent at 600 nm = 117,216
*A*_570_ = absorbance of test wells at 570 nm
*A*_600_ = absorbance of test wells at 600 nm
*E_red_*_570_ = E of reduced alamarBlue at 570 nm = 155,677
*E_red_*_600_ = E of reduced alamarBlue at 600 nm = 14,652
*C*_570_ = absorbance of negative control well (media, AlamarBlue Reagent, no cells, no treatment) at 570 nm
*C*_600_ = absorbance of negative control well (media, AlamarBlue Reagent, no cells, no treatment) at 600 nm

For the PI/Hoechst assay, after treatment for 24 h, propidium iodide (PI) and Hoechst 33,342 (Invitrogen-Fisher Scientific, Ilkirch Graffenstaden, France) were added at 1 µg/mL final concentration directly in each well. After a 30 min incubation, analysis was performed with ImageXpress Pico (Molecular Devices, San Jose, CA, USA) to count the number of live cells.

### 2.11. Firefly Luciferase Inhibition Assays

The Firefly luciferase inhibition assays were performed as described by Poutiainen et al. [30]. The purified Firefly luciferase enzyme was purchased from Sigma-Aldrich (Saint Quentin Fallavier, France). The Firefly luciferase was diluted to 0.1 µg/mL in PLB. 30 µL of the luciferase solution and 1.2 µL of each molecule were incubated at 22 °C for 20 min. Then, 10 µL was transferred to white 96-wells plate and 30 µL of LARII was added before measuring luciferase activity. The Firefly inhibitor β-naphthoflavone (1 µM) was used in the same conditions as a positive control of this assay. PLB was used as a blank sample to measure the background signal. Control samples (vehicle: DMSO, ethanol, nonane or water-exposed luciferase) were used to normalize the results.

### 2.12. Statistics

All calculations in part II were performed using Prism (GraphPad, San Diego, CA, USA) Assays were performed in six independent trials and statistically validated in relation to the control. *t*-test or one-way ANOVA were applied for each assay, depending on the number of conditions. If the requirements for the normality test were not fulfilled, Wilcoxon or Friedman tests were applied.

## 3. Results

### 3.1. Transcriptomic and Metabolomic Profiling of C. elegans WT vs. ahr-1(ia03) Mutant

#### 3.1.1. Whole *C.* *elegans* HR-MAS NMR Spectroscopy: The Inactivation of the AHR-1 Receptor Impacts Metabolism

**Figure 1:**
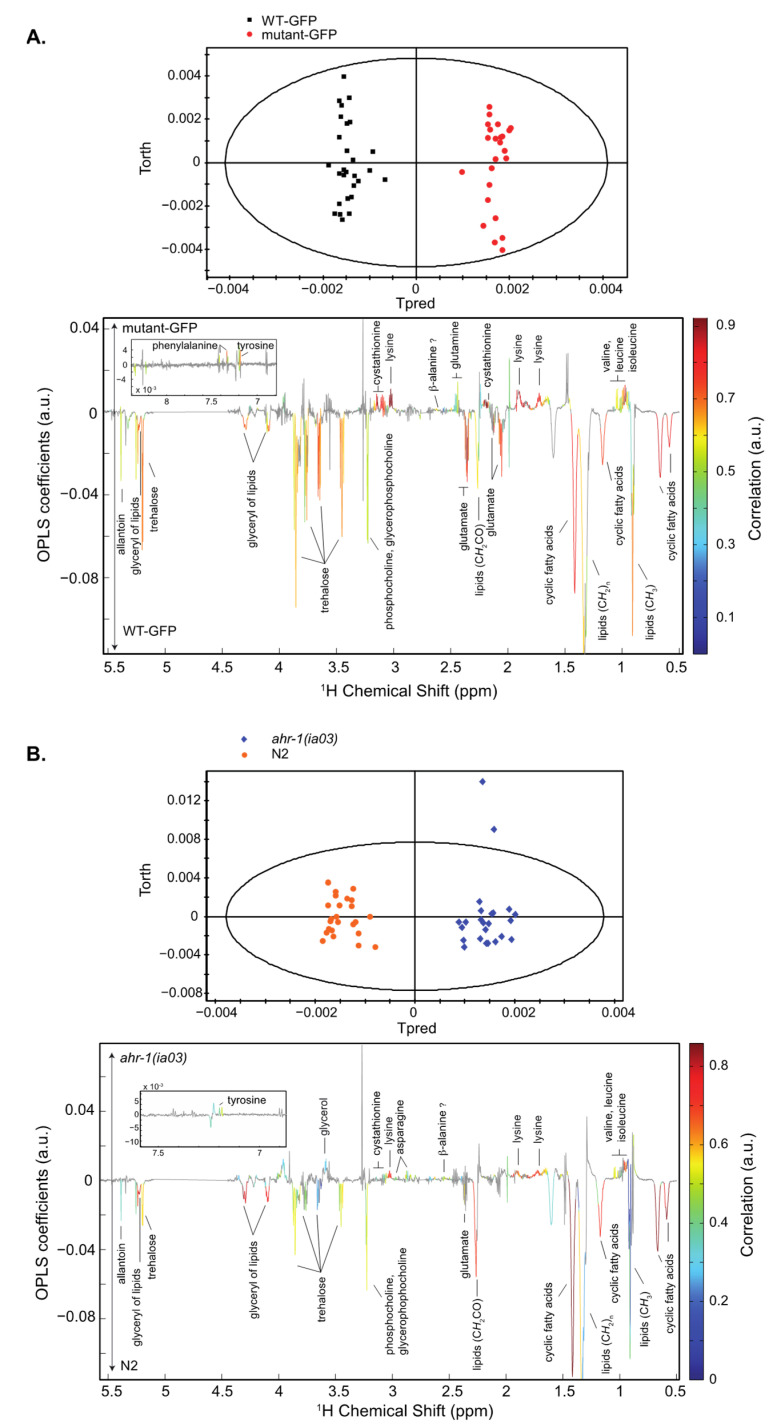
OPLS model discriminating WT-GFP and mutant-GFP (**A**) or N2 and *ahr-1*(*ia03*) (**B**). Top: Score plots. Bottom: Loading plots. (**A**) R2 = 0.783, Q2 = 0.973. mutant-GFP is associated with high levels of phenylalanine, cystathionine, lysine, b-alanine, glutamine, tyrosine, valine, leucine and isoleucine, and low levels of allantoin, trehalose, phosphocholine and glycerophosphocholine, glutamate, cyclic fatty acids and glyceryl of lipids, by comparison to WT-GFP. (**B**) R2 = 0.903, Q2 = 0.93. *ahr-1*(*ia03*) is associated with high levels of phenylalanine, glycerol, betaine, cystathionine, lysine, asparagine, tyrosine, valine, leucine and isoleucine, and low levels of allantoin, trehalose, glycerophosphocholine, phosphocholine, cyclic fatty acids and glyceryl of lipids, by comparison to N2.

Our omics experiments (transcriptomics and metabolomics) were parallelly designed. However, as our transcriptomics experiments involved a purification step of neurons expressing AHR-1, using GFP as a biomarker (under control of the *pmec3* promoter which regulates AHR-1 expression), we performed a metabolic analysis using two types of control nematodes (N2 and WT-*pmec3*::GFP, compared to the *ahr-1* mutants, *ahr-1(ia03)* and mutant-GFP, Figure 1 presents the NMR metabolic analysis).

Loss-of-function of the *ahr-1* gene induces several metabolic changes in *C. elegans* impacting amino-acid and carbohydrate levels as well as fatty acid metabolism. These metabolic perturbations were consistently observed in two *C. elegans* strains (which express or not a *gfp* gene reporter). Furthermore, the metabolic signature of *ahr-1* mutants, characterized by high levels of valine, isoleucine, leucine, lysine, cystathionine and tyrosine and low levels of oxidized and cyclic fatty acids, glycerophosphocholine and phosphocholine, shares great similarities with the metabolic signature of young adult wild-type worms compared to the adult ones [31]. These results suggest that AHR-1 is involved in the regulation of several endogenous metabolic pathways with possible links with aging and developmental processes.

#### 3.1.2. Global Gene Expression Profiles Are Altered in *C.* *elegans* ahr-1-Expressing Cells

**Figure 2:**
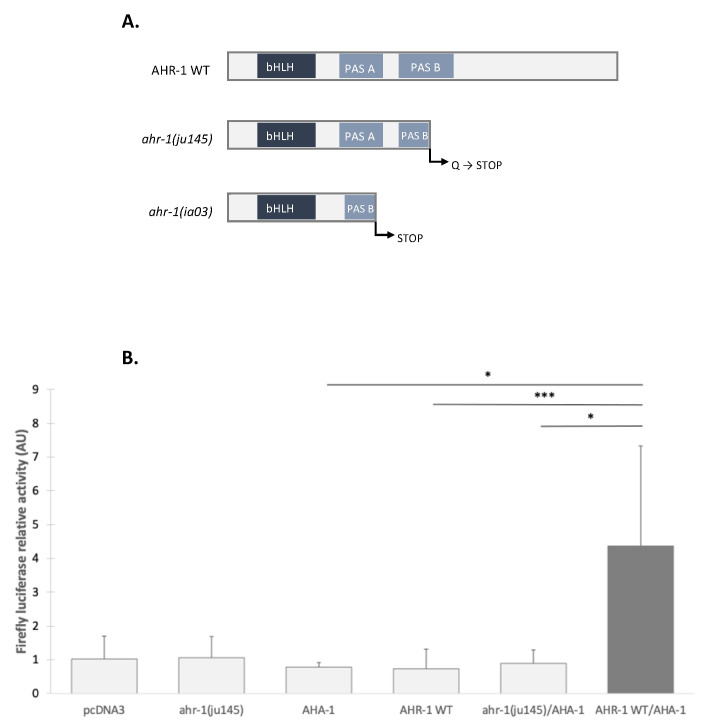
Requirement of AHR-1 and AHA-1 for transcriptional activity. (**A**) Structure of AHR-1 WT, *ahr-1(ju145)* and *ahr-1(ia03)* proteins. The 602 amino acids AHR-1 WT protein is truncated in *ahr-1*(*ju145)* as a result of a C to T point mutation that leads to a stop codon (Huang et al., 2004). The *ahr-1(ia03)* mutant allele contains a 1517 bp deletion from 205 bp 5′ of exon 4 to 30 bp 5′ of exon 8 that results in a frameshift and a premature stop codon (Qin and Powell-Coffman, 2004). (**B**) The requirement of the two partners is examined in the screening model. The Cos-7 cells transfection is carried out in different conditions with 5 ng/well of *ahr-1* (WT or mutants) and *aha-1* plasmids and without FBS: either with the empty vector (pcDNA3) or with only one of the two partners (AHA-1, *ahr-1(ju145)* or AHR-1 WT) and with the two partners (*ahr-1*(*ju145)*:AHA-1 and AHR-1 WT:AHA-1). Six independent experiments were performed in duplicate, error bars represent SD. Friedman test with Dunn’s multiple comparisons post-test * *p*-value < 0.05, *** *p*-value < 0.001.

To characterize which transcripts are regulated by AHR-1 in *C. elegans*, we isolated the cells expressing normally *ahr-1* in the nematode. To this aim, we generated two types of transgenic strains expressing the green fluorescent protein (GFP) under the transcriptional control of *mec-3* (*pmec-3*::GFP); MEC-3 is a transcription factor, expressed in specific neurons, which positively regulates the expression of AHR-1. Therefore, we used one strain expressing *pmec-3*::GFP in an otherwise wild-type background, while the other strain was the result of a cross between *pmec-3*::GFP and the *ahr-1(ia03)* mutant carrying a 1517 bp deletion that results in a frameshift and a premature stop codon (see Figure 2A for description of the mutation). GFP cells were isolated from both strains (WT: *pmec-3*::GFP vs. mutant: *pmec-3*::GFP;*ahr-1(ia03)*) using fluorescent activated cell sorting (FACS). RNA from both cell types were extracted, amplified, and analyzed using a *C. elegans* array from Agilent (for more details, see STAR Methods).

In total, 95 genes were down-regulated, and 76 genes were up-regulated in the *ahr-1::gfp* mutants, compared to the wild-type GFP, with cut-off values (a fold change ≥ 1.5 and a *p*-value < 0.05). The number of down-regulated genes was higher than the number of up-regulated genes in the *ahr-1* mutants, in line with previous microarray or RNASeq data [24,25].

Among the most down-regulated genes were several genes encoding guanylate cyclases (gcy) (*gcy-35*, *gcy-36*, *gcy-34*, *gcy-32*, *gcy-33*, *gcy-37*, *gcy-17*), the neuropeptide-like-protein gene (*npl-20*) and an FMRF-like peptide family gene (*flp-8*). Interestingly, three CYPs (*cyp-13A10*, *cyp-13A1* and *cyp-34A3*, Table 1) were also identified in our under-expressed genes in *ahr-1* KO neurons. Several genes encoding proteins involved in the synthesis of fatty acids (*elo-1* and *fat-2*) were among the up-regulated genes, which is consistent with the observed increase in the proportion of fatty acid C20:3n6 in *ahr-1* mutant L4 worms [24]. The ELO-1 protein (elongation of very long chain fatty acids protein) catalyzes the synthesis of C20:3n6 from C18:3n6.

We next used quantitative RT-PCR (using the same mRNAs extracted from *ahr-1* wild type and mutant worms) to confirm and better quantify the microarray data. Seven genes were tested (including *gcy-32*, *gcy-33*, *gcy-35*, *gcy-36* and *cyp-13A1*, *cyp-13A10*) and their down-regulation identified using microarrays was confirmed (Supplementary Data Figure S1).

In order to analyze the potential impact of AHR-1 on regulatory pathways, we used several methods including Gene Set Enrichment Analysis (GSEA). The results highlighted several processes that may be altered in the mutant animals as compared to their wild-type counterpart (Table 2). In mutant animals, gene sets associated with nervous system function were significantly depleted and functions associated with major metabolic pathways (fatty acid metabolism, oxidative phosphorylation, and glycolysis) were significantly enriched (Supplementary Data Figure S2).

### 3.2. Identification of AHR-1 Activity and Modulators In Vitro in Cos-7 Cells

Since several CYPs appeared to be induced by AHR-1, this led to reconsidering the functions of this protein in *C. elegans*, because it suggests that it may be involved in sensor activities, in line with the biological function of the vertebrates AhRs (which regulate the expression of the CYP1 family).

It is well known that in vertebrates, the AhR requires its nuclear partner ARNT to bind xenobiotic responsive elements (XRE) [32]. In *C. elegans*, AHR-1 and AHA-1 interact together to bind the same XRE as in mammals [17]. In silico analysis identified potential XREs (data not shown) in the promoters of two of the CYPs functionally linked to AHR-1 (*cyp-13A1* and *cyp-13A10*, the last one *cyp-34A3* is a pseudogene). In order to identify potential regulators of the AHR-1 function in *C. elegans*, we designed a reporter system assay using a highly sensitive natural promoter containing several functional XREs (human *cyp1A1* promoter) and two constructs allowing the reconstruction of a functional heterodimer AHR-1/AHA-1 in Cos-7 cells: these cells are commonly used for protocols using transient AhR-screening procedures due to their lack of a functional endogenous AhR pathway: Cos-7 cells have no detectable endogenous AhR [33], a small amount of ARNT [34] and no CYP1A1 activity [35]. We used this cell line from the Green Monkey *Chlorocebus sabeus* as: (1) no commercial *C. elegans* cell line is currently used; and (2) the use of primary neurons from the nematode would be difficult considering the small number of cells expressing *ahr-1*. Cos-7 cells are widely used for AhR screening experiments of different species [36,37,38].

#### 3.2.1. Optimization of the in vitro System

To confirm if the system works in the Cos-7 cells, we transfected cells with *ahr-1* and *aha-1*-expressing plasmids, alone or together and compared those conditions with the ones involving an empty vector as control (pcDNA3). Transfection of either *ahr-1* or *aha-1* alone does not lead to a significant increase of the luciferase activity and confirm the absence of endogenous AhR activity in Cos-7 cells. The only condition displaying a significant luciferase activity of the Firefly is the presence of both partners (AHR-1 and AHA-1, Figure 2B).

**Figure 3:**
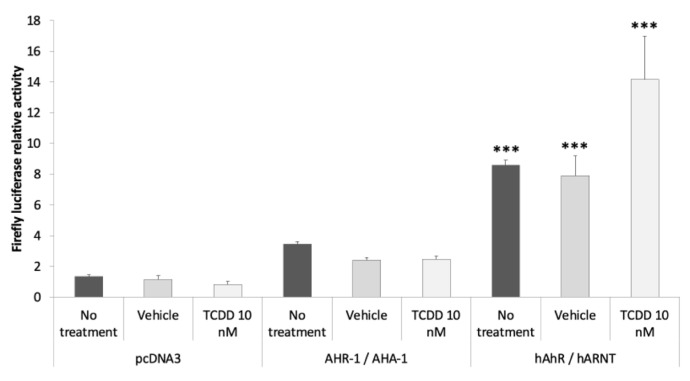
Comparison between Cos-7 cells transfected with *C. elegans ahr-1* or human *ahr*. Cos-7 cells were transfected with the empty vector (pcDNA3) or with *C. elegans ahr-1*/*aha-1* or with human *ahr*/*arnt*. After transfection and culture for 24 h in 0% FBS medium, cells were treated for 24 h with 10 nM of TCDD. The relative activity represents the Firefly measurement normalized to the Renilla measurement for each condition. Three independent experiments were performed in duplicate, error bars represent SD. ANOVA followed with comparison test of each column with the control column (pcDNA3—No treatment) *** *p*-value < 0.001.

We also carried out RT-qPCR on cells treated with TCDD (not transfected) since we expected that this dioxin should be a ligand of the Green Monkey AhR (considering the conservation of TCDD binding in the whole vertebrate lineage). Again, we did not observe any increase in the mRNA expression of the prototypical target gene *cyp1a1* in Cos-7 cells (Supplementary Data Figure S3), compared to the strong induction observed in the human HepG2 cell line. Finally, to check that Cos-7 cells could represent a good model to test the activity of the AhR, we compared the potentiality of AHR-1 and of the human AhR. Experiments were performed with the empty vector, AHR-1/AHA-1 or human AhR/ARNT, and cells were treated with 10 nM of TCDD. We observed a basal activity of the human AhR/ARNT without treatment. Treatment with TCDD resulted in a 1.79-fold activation of human AhR compared to the vehicle when there is no effect on AHR-1 (Figure 3). There is no strong activation in our system but it is well known that Cos-7 cells transfected with vertebrate AhR (human, fish, bird, etc.) do not have a strong expression of the Firefly luciferase under the control of a promoter containing XREs as opposed to other human cell lines [39,40,41]. Moreover, to amplify the activity of a transcription factor in transfected mammalian cells, the VP16 transactivation domain is commonly fused with the gene of interest. We then added the VP16 transactivation domain to *ahr-1* and *aha-1*-expressing vectors because the lack of information about coregulators of *C. elegans* AHR-1 does not allow us to check if Cos-7 cells possess the partners to modulate the transcriptional activity of AHR-1/AHA-1. We noticed a weak activation of AHR-1 in the Cos-7 cells despite adding the transcriptional activity domain (TAD) of VP16 to the C-terminal region of AHR-1. It could suggest a low sensitivity to our system, and then the modulators identified might in fact be strong potentiators in vivo.

In conclusion, the transfection of *ahr* and *arnt*-expressing vectors in our model is functional and responsive to human ligands suggesting that *ahr-1*/*aha-1* transfection could be symmetrically used to pinpoint AHR-1 nematode modulators.

#### 3.2.2. The Transcriptional Activation Domain of AHR-1 Is Required for Gene Expression

AHR-1 shares biochemical properties with its mammalian cognate; however, some structural differences were noted. The receptor contains a transcriptional activity domain (TAD) [17] but it lacks the Q-rich domain [42]. To confirm that TAD is required for gene expression, we used the *ahr-1(ju145)* mutant alone or with AHA-1 in the Cos-7 cells. None of these conditions activated transcription of the luciferase reporter, indicating that the transcriptional activity domain of AHR-1 is essential for gene expression and that AHA-1 is not sufficient (Figure 2). So the Q-rich domain may not be necessary in *C. elegans*: the coregulator recruitment could be different than the one in vertebrates and might involve another structure than the Q-rich domain (it is known in vertebrates that some coregulators such as CoCoa or GaC63 bind the bHLH/PAS domain) [43].

#### 3.2.3. AHR-1 Basal Activity in Cos-7 Cells

**Figure 4:**
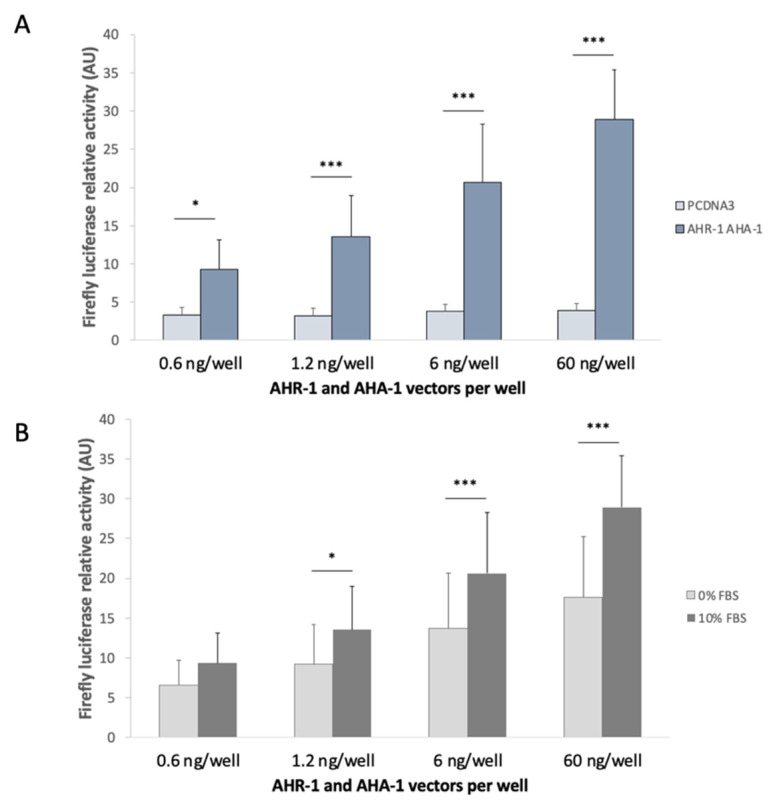
AHR-1 basal activity in Cos-7 cells. Different plasmid concentrations and FBS conditions were tested during the screening model optimization. (**A**) Comparison between the transfection of the empty vector (pcDNA3) and the two partners (*ahr-1* and *aha-1* plasmids) in Cos-7 cells cultured with 10% FBS. (**B**) Comparison between the transfection of *ahr-1* and *aha-1* plasmids in Cos-7 cells cultured either in 0% FBS or in 10% FBS. The relative activity represents the measurement of the Firefly activity normalized to the Renilla measurement for each condition. Six independent experiments were performed in duplicate, error bars represent SD. ANOVA with Bonferroni’s multiple comparison post-test * *p*-value < 0.05, *** *p*-value < 0.001.

During the optimization of the screening model, we carried out transfection experiments with different amounts of plasmids: from 0.6 to 60 ng/well for *ahr-1* and *aha-1* or for the empty vector (pcDNA3). Compared to pcDNA3, we observed an increase expression of our reporter suggesting a basal activity of the heterodimer AHR-1/AHA-1 without any treatment of the Cos-7 cells; this depends on the amount of *ahr-1* and *aha-1* vectors transfected (Figure 4A), suggesting that the heterodimer is active either without any modulator, or with modulators present in the cell culture medium (even if we limited the presence of potential activators in this medium). We hypothesized that this activity of AHR-1 could be related to the presence of an activator in fetal bovine serum (FBS). Therefore, we carried out an experiment using the same protocol as previously but removing the FBS together with the transfectant (4 h after cell transfection). We still observed a basal activity of the receptor but lower than the one observed using 10% FBS in the cell medium (Figure 4B). These results suggest that some compounds in FBS can increase AHR-1 activity. This could be due either to the activation of the receptor, or by elevating the amount of AHR-1 protein (by increasing its synthesis or decreasing its degradation).

#### 3.2.4. Identification of AHR-1 Positive and Negative Modulators

To guide the choice of molecules used in the screening test, we firstly chose to test some of the human AhR agonists or antagonists such as environmental, dietary, or endogenous compounds. We also tested bacterial compounds or virulence factors which are part of the natural environment of *C. elegans* (and are human AhR ligands).

First, we verified that such molecules did not exert any toxicity in our model. Two cell viability assays have been carried out: Alamar Blue and Neutral Red (Supplementary Data Table S1), for each molecule identified subsequently as a modulator of AHR-1. A PI/Hoechst assay was performed for pyocyanin especially, which interferes with the other assays, by its oxidative properties. Second, we performed an inhibitory enzymatic assay of the Firefly luciferase (Supplementary Data Table S2) to confirm that the resulting bioluminescence is related to an activation of AHR-1 and not a direct effect of the molecules tested on the enzyme (because some chemicals identified as AhR ligands, such as β-naphthoflavone or resveratrol, also inhibit luciferases, and this effect is often neglected) [44,45]. These compounds can act as competitive or non-competitive inhibitors of the catalytic activity of the enzyme and are a critical problem when using Firefly luciferase as the reporter gene [46]. Finally, molecules were tested on cells transfected with *ahr-1/aha-1* or with the empty vector pcDNA3, to confirm that the bioluminescence observed is only due to the transcriptional activity of AHR-1/AHA-1 and not to another mechanism (the level of bioluminescence with pcDNA3 is the same as the background for all modulators, data not shown).

In invertebrates, AhR was thought not to bind any prototypical ligands of the vertebrate AhR such as TCDD or β-naphthoflavone [17,18,47,48]. As seen in Figure 4, we confirmed the absence of direct or indirect activation of AHR-1 by TCDD 10 nM. We could not confirm the β-naphthoflavone because this molecule is a potent inhibitor of the Firefly luciferase ([44]; Supplementary Data Table S2). Tryptophan and some of its metabolites, as well as polyphenols such as resveratrol or quercetin, known to modulate the mammalian AhR, did not modulate AHR-1 activity (see Supplementary Data Table S3 for tested molecules with no significant effect on AHR-1 activation).

**Figure 5:**
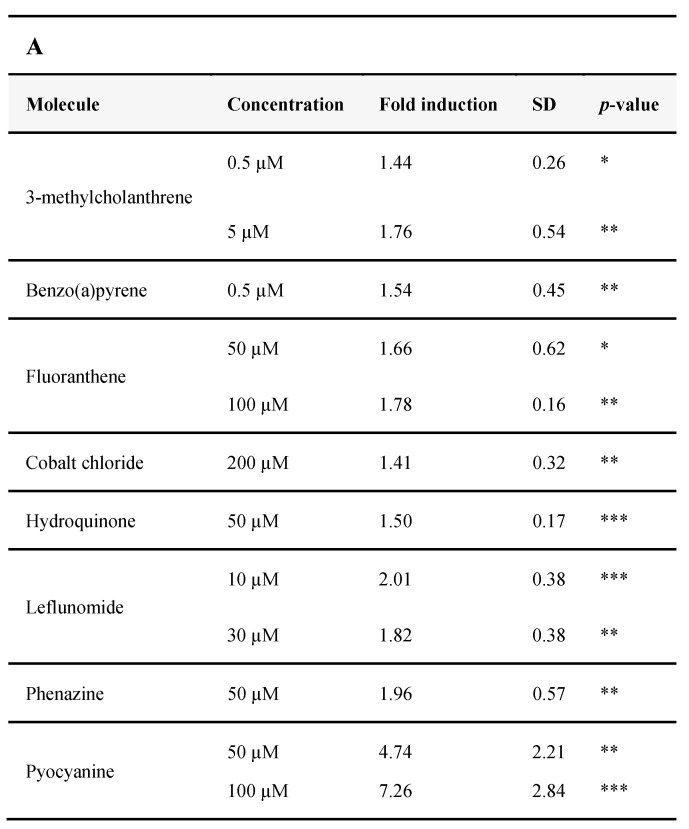

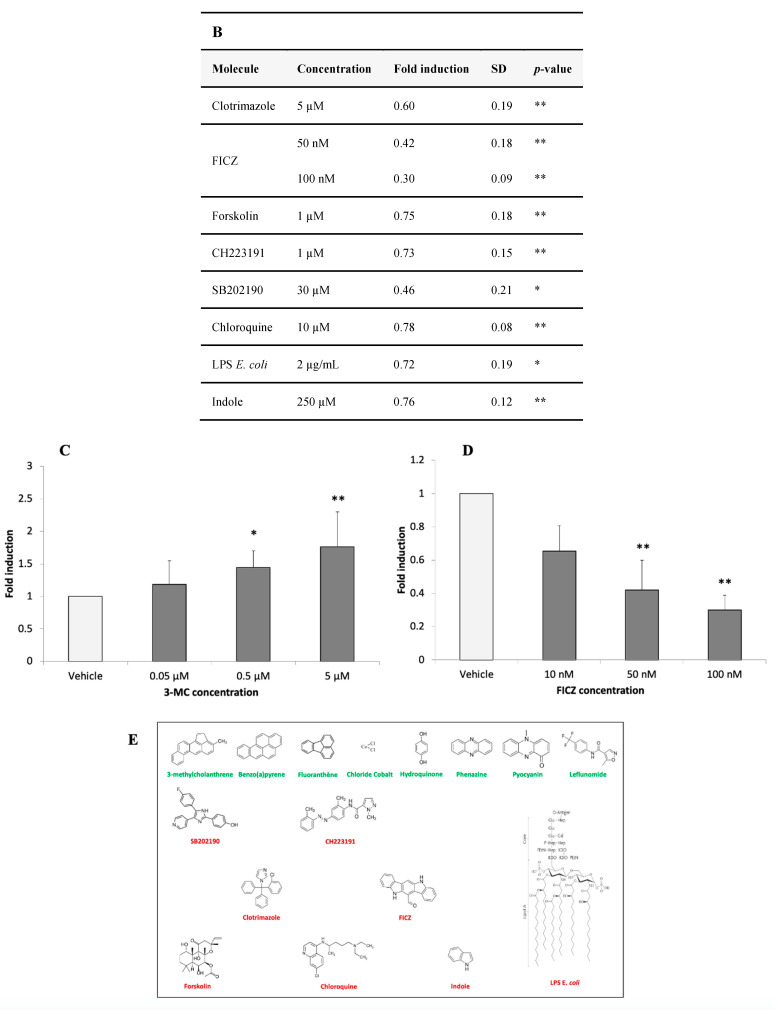
Summary of significant AHR-1 modulators in Cos-7 cells. Positive (**A**) and negative (**B**) modulator’s concentrations are represented with the dependent-AHR-1 fold induction of Firefly luciferase. Example of a positive (**C**), and negative (**D**) modulator dependent concentration effect on AHR-1 activity. Six independent experiments were performed in duplicate, fold induction is standardized to the vehicle which is 1. Statistical significances relative to the vehicle were examined: * *p*-value < 0.05; ** *p*-value < 0.01; *** *p*-value < 0.001. (**E**) Molecular structure of the positive (green) and negative (red) modulators of AHR-1.

Interestingly, some PAH such as 3-methylcholanthrene (Figure 5C), benzo(a)pyrene or fluoranthene, can positively modulate AHR-1 activity (Figure 5A). An induction of AHR-1 is observed with the immunomodulatory drug leflunomide which is an AhR agonist in zebrafish [49], at higher concentrations than those already used (Brinkmann et al., submitted along with this study). Some negative modulators were also identified (Figure 5B). For example, the most potent endogenous ligand of human AhR, FICZ shows surprising results in *C. elegans* AHR-1, representing the strongest concentration-dependent negative modulator, identified until now (Figure 5D).

To investigate the modulatory roles of the *C. elegans* natural environment, we screened bacterial compounds (the main source of food for the nematode) and identified phenazine as a positive modulator. We also found the highest activation of AHR-1 in our system with pyocyanin (fold induction of 7.56 for a concentration of 100 µM). On the other hand, lipopolysaccharide (LPS) from *Escherichia coli* was characterized as a negative modulator. However, a pellet of bacteria (*E. coli*) or flagellin (the major compound of the bacterial flagella) did not have any effect.

AHR-1 was described to promote aging in *C. elegans* [50]. Moreover, the involvement of indole in the extended lifespan of *C. elegans* is known as acting via AHR-1 with no mechanistic hint [51]. We tested indole and observed a decrease of AHR-1 activity at the concentration of 250 µM. Then, this result could provide a mechanistic link with the increase of *C. elegans* lifespan by reducing AHR-1 activity.

**Figure 6:**
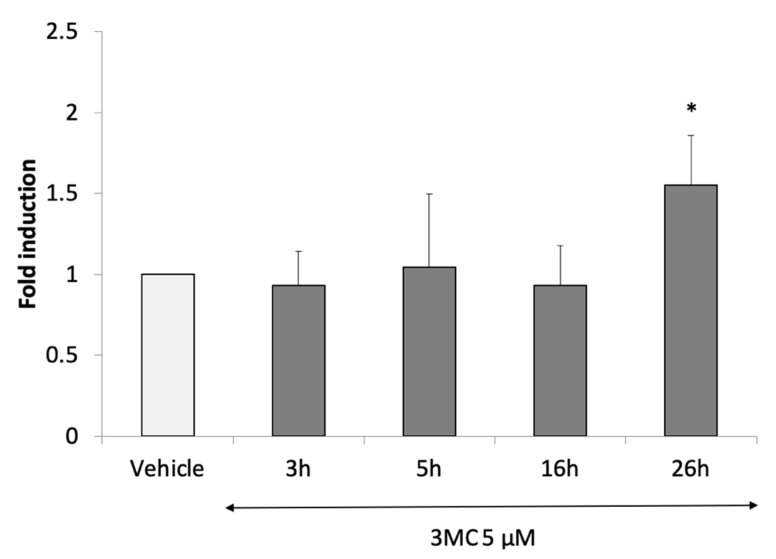
Time course of 3MC, a positive modulator of AHR-1 in Cos-7 cells. After transfection and culture for 24 h in 0% FBS medium, cells were treated for 24 h with 3-methylcholanthrene 5 µM. After 3 h, 5 h, 16 h and 26 h of treatment, cells were lysed, and Firefly luciferase luminescence was read. Six independent experiments were performed in duplicate, error bars represent SD, fold induction is standardized to the vehicle which is 1. Statistical significances relative to the vehicle were examined: ANOVA with Bonferroni’s multiple comparison post-test, * *p*-value < 0.05.

The induction of target genes by the human AhR could be observed after long periods of treatment (24 h after treatment with 3-methycholanthrene, 3MC) [52]. We then performed a short-period kinetic experiment using 3MC and confirmed that the modulation of the Firefly luciferase activity caused by AHR-1 occurred after only 24 h of treatment and not before (Figure 6). However, because of the limitation of our system (the cells die after three days of protocol), we cannot observe how many times the Firefly luciferase activity is increased and from when it decreases.

## 4. Discussion

The primary goal of our study was to identify new endogenous functions of the AhR ortholog of *Caenorhabditis elegans*, AHR-1, by the comparison of mRNA expression profiles in purified neurons between *C. elegans ahr-1* WT and *ahr-1(ia03)* mutant models. In the nematode, RNA profiling from whole animals has been applied successfully in previous transcriptomic studies in search for differences in gene expression (male vs. hermaphrodite animals, between different developmental stage…). To date, our study is the first one performed in *C. elegans*, which utilized isolated cells expressing *ahr-1* in wild-type animals (and their counterparts from knockout animals). For genes that are expressed only in a subset of neurons, which is the case of *ahr-1*, the identification of mild modulation is difficult using whole-worm-extracted RNA, containing nearly 1000 cells. Furthermore, gene expression can vary greatly between developmental stage in the nematode, as revealed by the differentially expressed gene lists between *ahr-1* WT and mutant conditions in two global transcriptomic analysis using whole worm at L4 and L1 stages [24,25]. We have thus chosen to use fluorescence activated cell sorting to isolate AHR-1::GFP-tagged neurons prior to mRNA extraction and microarray analysis. These findings, in which *npr-1*, *nlp-20* and *gcy* genes were found to be under-expressed to a great extent in *ahr-1* KO neurons, extend previous studies demonstrating the key role of this receptor in the regulation of social aggregation behavior of the nematode, through the guanylate cyclase and neuropeptide family genes. Indeed, Qin and Powell-Coffman have shown that in *ahr-1* mutant, two markers of URX differentiation (*gcy-32* and *npr-1*) are expressed at markedly reduced levels compared to wild-type animals; and a genetic inactivation of URX neurons suppressed *npr-1*-mediated social behavior [21,53].

In addition, we identified three putative functions as being predominantly affected by *ahr-1* knockout: dysregulation of the nervous system, impact on the oxidative and fatty acid metabolisms. These functions have been described in vertebrate species and might be shared in the bilaterian phylum (e.g., invertebrates and vertebrates). In vertebrates, the AhR loss-of-function in mice causes myelin defects that affect the peripheral nervous system and is associated with a horizontal pendular nystagmus, an ocular pathology [13,14,16] and this myelination deregulation has been also characterized in the human peripheral nervous system [15]. Moreover, AhR KO mice reveal a disruption in oxidative metabolism such as a down-regulation of genes involved in oxidative phosphorylation [54] or accumulation of mitochondrial oxidative phosphorylation intermediates. These AhR-deficient mice displayed decreased fatty acid β-oxidation or fatty acid uptake enzymes, and a reduction of PPAR-α expression involved in the regulation of fatty acid metabolism [55,56]. The potential impact on the fatty acid metabolism is coherent with our findings using whole-organism HR-MAS NMR spectroscopy to characterize metabolic changes induced by AHR-1 inactivation. This is also the case of fatty acid composition which is altered in *ahr-1* mutant worms [24].

In our microarray data, three CYPs (*cyp-13A10*, *cyp-13A1* and *cyp-34A3*, Table 1) were also found to be under-expressed in *ahr-1* KO neurons. Our results point out differences in CYP expression compared to the one performed on whole animals (Brinkmann et al., submitted along with this study), and could reveal a neuronal specific phenotype. Regarding the results, we cannot state that up-regulation or down-regulation of mRNA are directly linked to transcriptional effects but the identification of these three CYPs was intriguing, suggesting that a potential AHR-1/CYP pathway might exist in *C. elegans*, similarly to the classical AhR pathway in vertebrates. The reduced expression of CYP genes observed here *in ahr-1* KO neurons are in line with previous studies that demonstrated that AhR promotes aging phenotypes in human, mice, and *C. elegans*. AhR deficiency and the down regulation of its target genes were observed as beneficial during aging [50]. Indeed, as shown by our metabolic analysis, a loss-of-function mutation in the *ahr-1* gene induces strong metabolic changes in *C. elegans* and this metabolic signature shares great similarities with the metabolic signature of young adult wild-type worms compared to the adult ones. Furthermore, an FMRF-Like Peptide family gene (*flp-8*) was also identified in our top under-expressed gene in *ahr-1* KO neurons. A recent study has highlighted the role of neuronal *flp*-signaling in regulating lifespan in *C. elegans* [57]

To date, no ligand was identified to activate invertebrate AhRs. Spineless, the AhR ortholog of *D. melanogaster*, was found to be constitutively active *in vitro*, and could explain its inability of ligand activation [58]. Our results are consistent with the hypothesis that AHR-1 activity is inducible in vitro [17] and not constitutive like Spineless.

We refer in this study to AHR-1 modulators and not ligands because the direct binding to the transcription factor is not yet confirmed. In addition, all the molecules we found as AHR-1 modulators in our in vitro system should be back verified in vivo in the worm to confirm our results. We observed a positive or negative modulation of AHR-1 transcriptional activity with compounds from various origins, having different chemical structures. One possibility could be that some of them may not be able to bind the putative ligand binding domain of AHR-1. The indirect modulation by these modulators could be, for example, related to the influence on a cellular parameter such as oxidative stress that plays a role in human AhR activation [59]. In addition, the modulation of AHR-1 could be linked to the activation of other proteins such as kinases (protein kinases A and C or p38 MAPK) involved in the phosphorylations and activation of AhR in vertebrates [60,61]. So far, we did not provide any evidence that invertebrate AhR can bind ligands, and activation of AHR-1 by our modulators could be indirect.

The most interesting positive modulator we found is pyocyanin. This compound is derived from phenazine which is a virulence factor from *Pseudomonas aeruginosa* (a lethal pathogen for *C. elegans*). Considering that the main source of food for the nematode is microorganisms such as bacteria, it could suggest that AHR-1 can act as an environmental sensor, for example to prevent toxicity linked to the food intake. One hypothesis about AhR functions is that its physiological roles appear before its adaptive roles. This was suggested because AhR in invertebrates is unable to bind dioxins and other prototypical ligands found in vertebrates. The adaptive role as a chemical sensor, by regulating xenobiotic metabolizing enzymes, was thought to be an innovation that appeared in the vertebrate lineage. However, some studies on invertebrate AhR in mollusks, rotifers or arthropods showed that exposure to polycyclic aromatic hydrocarbons (PAHs), such as BaP, leads to an increase in AhR, ARNT and some xenobiotic metabolizing enzymes (CYP) mRNA expression [62,63,64,65,66]. We demonstrated that *C. elegans* AHR-1 activity is positively modulated by some PAHs and our transcriptomic results in neurons indicate that *cyp-13A1* and *cyp-13A10* expression is decreased in *C. elegans ahr-1(ia03)* mutants. Thereby, even if there is no direct evidence that invertebrate AhR can bind xenobiotics, a correlation could exist between an exposure to these chemicals and the induction of some metabolizing enzyme homologs known in vertebrates. This could be evidence of a signaling pathway involving the AhR that appears to be conserved in invertebrates.

## 5. Conclusions

Our study opened several key questions to fully understand the role of AHR-1 in *C. elegans* and, more generally, the functions of AhR in vertebrate and invertebrate species.

Taken together, our transcriptomic and metabolic findings extend previous studies and furthermore suggested a key involvement of AHR-1 by mediating the regulation of several endogenous pathways, such as neuronal and metabolic pathways, linked to development and aging in *C. elegans*. One interesting point is that AHR-1 appears to be involved in the expression of several CYPs, and suggests a potential sensor activity for AHR-1, even if it seems to be different or not as efficient as the vertebrate detoxification system. This hypothesis is reinforced by the fact that AHR-1 could be a direct or indirect sensor of environmental chemicals in *C. elegans*, including bacterial compounds. For the first time, the identification of a modulation of AhR transcriptional activity by chemicals in an invertebrate specie indicates similarities with vertebrates. However, further studies should be carried out in order to better understand whether the molecules identified are direct ligands or modulators acting indirectly such as through post-translational modifications. Moreover, the involvement of these modulators in physiological processes must be elucidated. For example, is there a conserved pathway in *C. elegans* from modulators to neurophysiology via AHR-1, as described in vertebrates?

Further detailed characterization of the newly identified targets from our transcriptomic analysis using wild-type and *ahr-1* mutants, treated or not with known xenobiotics or modulators identified in our Cos-7 model, might produce a strong fundamental impact in the AhR fields considering the lack of studies on neurons or on the functions of the unliganded receptor.

## Figures and Tables

**Table 1:** Gene expression profile in *ahr-1* mutants vs. wild-type animals. Top list of down-regulated and up-regulated genes in *ahr-1* mutant vs. WT *C. elegans*.

Gene Symbol	Gene Description	Fold Change KO/WT
gcy-35 npl-20 B0207.7 flp-8 gcy-36 K09E4.4 gcy-34 gcy-32 T09B9.5 cyp-13A10 agr-1 F18G5.1 C18C4.1 C05D9.4 kin-1 sra-21 C17F4.8 srw-129 hlh-26 Y57G11C.20 gcy-33 T05A7.6 hlh-14 T27C5.6 F14F9.4 gcy-37 F14F9.3 che-11 srh-261 clec-18	Guanylate CYclase Neuropeptide-Like Protein Predicted to have serine/threonine kinase activity FMRF-Like peptide Guanylate CYclase Orthoilog of human NAGLU (N-acetyl-alpha-glucosaminidase) Guanylate CYclase Guanylate CYclase T09B9.5 CYtochrome P450 family AGRin (synaptic protein) homolog F18G5.1 glb-5 (GLoBin related) C05D9.4 Protein kinase Serpentin receptor, class A (alpha) Predicted to encode a protein with BTB/POZ domain Serpentin receptor, class W Helix Loop Helix Uncharacterized protein Guanylate CYclase Predicted to have serine/threonine kinase activity Helix Loop Helix F14F9.4 Guanylate CYclase F14F9.3 Abnormal CHEmotaxis Serpentin Receptor, class H C-type LECtin	0.07 0.11 0.15 0.20 0.28 0.29 0.29 0.30 0.35 0.37 0.37 0.41 0.42 0.43 0.47 0.48 0.49 0.50 0.50 0.52 0.52 0.52 0.53 0.53 0.54 0.54 0.55 0.55 0.55 0.55
Ribonuclease T27D12.1 C12D5.3 nspc-12 Y62H9A.2 nscp-10 C13A2.5 bus-12 fbxb-54 irg-5 clec-34 F22E5.8 T21C12.8 W06D4.3 str-208 oac-36 T21C12.8 R07E3.2 dnc-6 clec-116 pho-10 F07E5.7 C13A2.10 col-41 dpy-13 R13H4.8 T22B2.6 sqt-1 pgs-1 cnk-1	K10C9.3 T27S12.1 C12D5.3 Nematode Specific Peptide family, group C Y62H9A.2 Nematode Specific Peptide family, group C Predicted to have transferase activity, transferring glycosyl groups Ortholog of human SLC35D3 (solute carrier family 35 membre D3) F-box B protein Infection Response Gene C-type LECtin F22E5.8 T21C12.8 W06D4.3 Seven TM Receptor Predicted to have transferase activity R07E3.2 DyNactine Complex component Is predicted to have carbohydrate binding activity intestinal acid PHOsphatase Predicted to encode a protein with TRA-1 regulated domain Predicted to encode a protein with the methyltransferase FkbM domain Predicted tob e a structural constituent of cuticle DumPY:shorter than wild-type R13H4.8 T22B2.6 Ortholog oh human METTL24 and SCARA3 PhosphatidylGlycerophosphate Synthase Connector/eNhancer of KSR	1.75 1.76 1.76 1.77 1.78 1.80 1.88 1.89 1.94 1.94 1.99 2.06 2.06 2.12 2.16 2.21 2.27 2.32 2.51 2.52 2.60 2.70 2.80 2.83 3.67 4.13 4.32 4.44 4.81 10.64

**Table 2:** Gene set enrichment analysis of *ahr-1* mutant vs. WT phenotypes. GSEA shows the highest depleted and enriched gene sets in *ahr-1* mutant phenotype in comparison to the wild type one. NES: normal enrichment score. Gene sets are GO terms or from https://www.gsea-msigdb.org (accessed on 20 May 2022).

Gene Set Name (*C. elegans* Gene Only)	NES	*p*-Value	AhR KO Phenotype
Regulation of nervous system development	0.73	0.002	Depleted
Mecanosensory behavior	0.63	0.031	
Neuron migration	0.58	0.084	
Synaptic transmission	0.52	0.010	
Neurogenesis	0.51	0.035	
Learning and memory	0.48	0.115	
Fatty acid biosynthetic process	−0.78	0.008	Enriched
Glycolysis	−0.67	0.054	
Fatty acid metabolic process	−0.66	0.058	
Oxidative phisphorylation	−0.64	0.004	
Ribosome	−0.52	0.065	

## Data Availability

The microarray data can be accessed through the Gene Expression Omnibus accession no. GSE195728.

## Supplementary Materials

The following supporting information can be downloaded at: https://www.mdpi.com/article/10.3390/antiox11051030/s1, Figure S1: Fold change of ahr-1 KO / Wild type condition of selected genes as measured with real-time qPCR; Figure S2: Gene set enrichment analysis plot; Figure S3: mRNA relative expression level in different cell lines; Table S1: Cell viability assays; Table S2: Firefly luciferase inhibitory assay; Table S3: Tested compounds with no significant effect; Table S4: Primers used for the quantitative real-time polymerase chain reaction.
