# Supplementary material for: Identification of Modulators of the C. elegans Aryl Hydrocarbon Receptor and Characterization of Transcriptomic and Metabolic AhR-1 Profiles
Source: Antioxidants (Basel). 2022 May 23;11(5):1030. doi: 10.3390/antiox11051030 (PMC9137885; doi:10.3390/antiox11051030)
Supplement: Supplementary file 1 [file antioxidants-11-01030-s001.zip › antioxidants-1645299-supplementary.pdf]

Supplementary data and tables

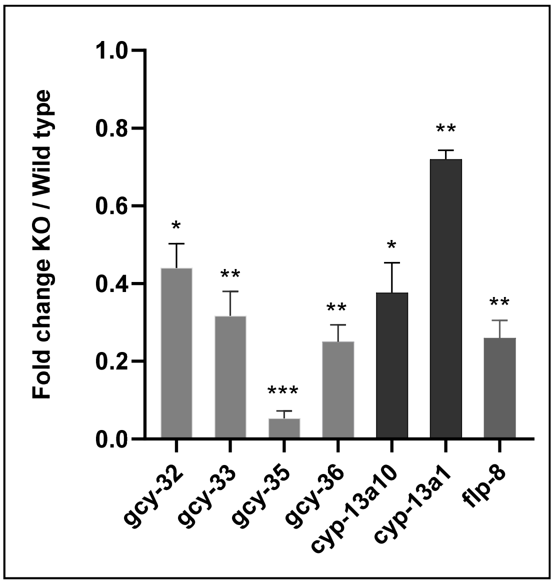

Figure S1. Fold change of *ahr-1* KO / Wild type condition of selected genes as measured with real-time qPCR. The qPCR was performed for three independent biological replicates. Values are expressed as mean ± s.e.m. t-test *p*-value * P < 0,05, ** P < 0,01, *** P < 0,001.

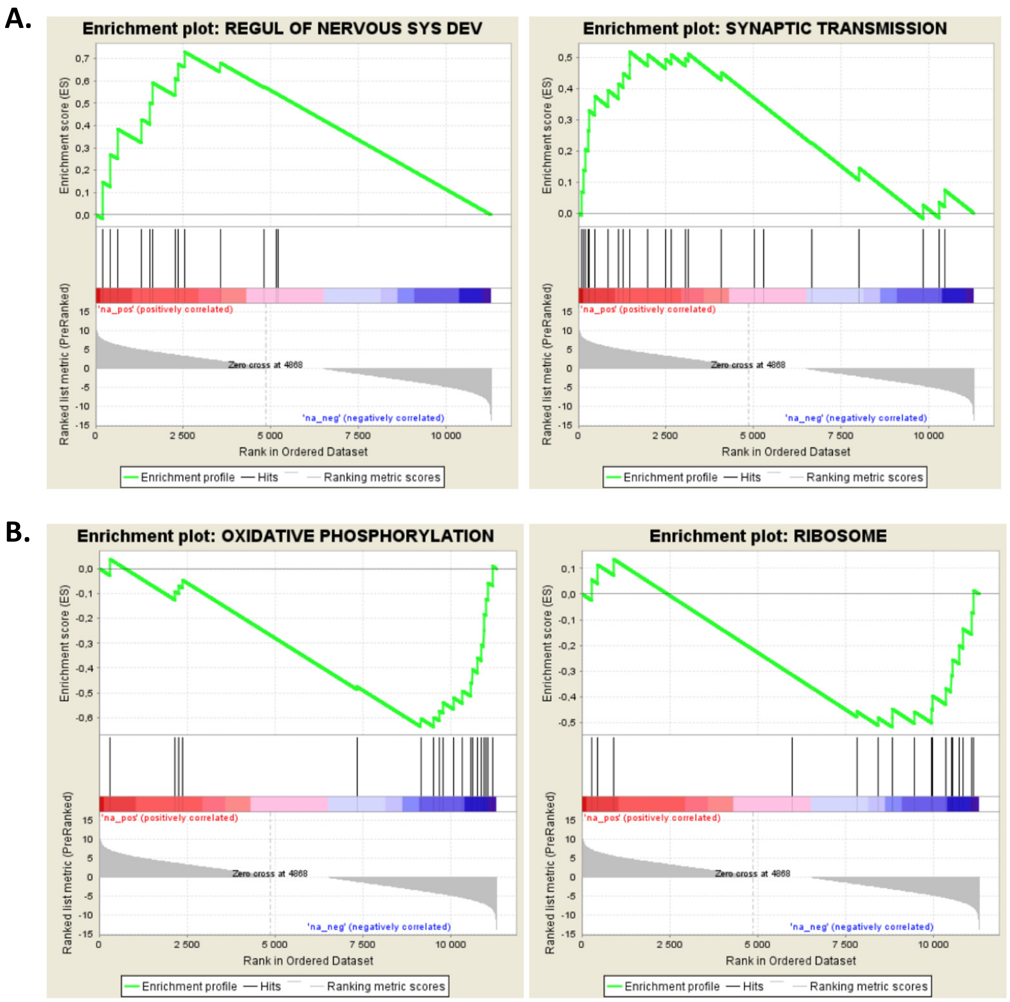

Figure S2. Gene set enrichment analysis plot. GSEA plot shows that (**A**) the most important depleted groups of genes found in *ahr-1* KO neurons belonged to nervous system functions and (**B**) that gene sets associated with oxidative phosphorylation and ribosome were significantly enriched in KO neurons (The same results were observed with fatty acid process and glycolysis). The data were grouped into wild type condition (na_pos) and KO condition (na_neg). The left half of each graph (red portion) shows the positive correlation with the AHR-1 wild type pattern.

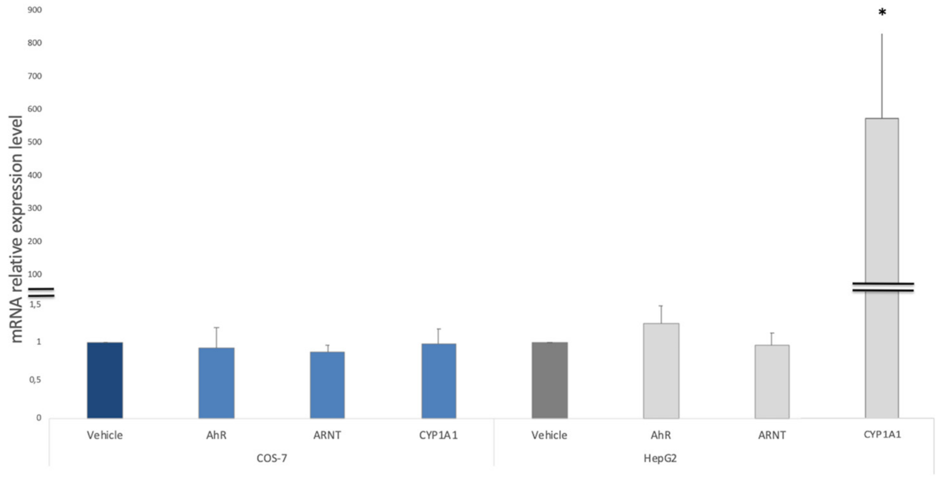

Figure S3: mRNA relative expression level in different cell lines. Cos-7 (blue) or HepG2 (gray, used as positive control) cells were treated with TCDD 24 hours before RNA extraction. mRNA expression levels were evaluated by RT-qPCR and normalized to the vehicle (nonane) which is 1 for each cell line. Four independent experiments were performed in duplicate, error bars represent SD. Statistical significances relative to the vehicle were examined: ANOVA with Dunnett's multiple comparison post-test, *p-value < 0,05.

Table S1: Cell viability assays. A) Neutral Red Uptake Assays and **B**) Alamar Blue Assays for each molecule tested on Cos-7 cells. **C**) Percentage of transfected cell viability compared to the non-transfected cells for the two assays. **D)** PI/Hoechst Assay. All assays were performed in six independent experiments. Alamar Blue assays without cells were performed in parallel to evaluate the percentage of Alamar Blue reagent reduction of each molecule. Statistical significances relative to the vehicle were examined: * p-value < 0,05; ** p-value < 0,01; *** p-value < 0,001

A

Solvent	Molecule	Concentration	% of cell viability	SD	*p*-value
DMSO	Hydroquinone	50 µM	111,11	0,22	
	3-methylcholanthrene	0,5 µM	104,25	0,08	
		5 µM	102,26	0,10	
	Benzo(a)pyrene	0,5 µM	107,74	0,20	
	Fluoranthene	50 µM	86,13	0,09	**
		100 µM	84,40	0,09	**
	CH223191	1 µM	118,25	0,11	*
	FICZ	50 nM	105,53	0,15	
		100 nM	96,25	0,04	
	SB202190	30 µM	88,84	0,19	
	Curcumin	100 µM	124,38	0,18	*
	Clotrimazole	5 µM	130,83	0,29	
	Indole	250 µM	110,30	0,05	*
	Forskolin	1 µM	101,97	0,21	
	Leflunomide	10 µM	104,02	0,08	
		30 µM	91,31	0,11	
H_2O	Cobalt Chloride	200 µM	107,11	0,10	
	LPS E. coli	2 µg/mL	109,39	0,09	*
	Chloroquine	10 µM	107,66	0,12	
Ethanol	Phenazine	50 µM	95,87	0,11	
Nonane	TCDD	10 nM	96,95	0,01	**

B

Solvent	Molecule	Concentration	% of cell viability	SD	*p*-value	% of Alamar Blue reduction without cells	SD	*p*-value
DMSO	Hydroquinone	50 µM	70,88	0,11	**	427,59	0,78	***
	3-methylcholanthrene	0,5 µM	99,41	0,05		133,57	0,16	***
		5 µM	101,57	0,13		143,21	0,20	***
	Benzo(a)pyrene	0,5 µM	88,31	0,09		140,16	0,20	**
	Fluoranthene	50 µM	63,85	0,06	***	89,55	0,12	
		100 µM	61,71	0,06	***	80,90	0,15	*
	CH223191	1 µM	100,43	0,07		85,58	0,23	
	FICZ	50 nM	98,52	0,07		102,12	0,14	
		100 nM	101,50	0,13		104,52	0,09	
	SB202190	30 µM	89,28	0,11		115,97	0,26	
	Curcumin	100 µM	97,61	0,07		113,83	0,17	
	Clotrimazole	5 µM	95,53	0,09		116,24	0,22	
	Indole	250 µM	96,10	0,04		80,44	0,13	*
	Forskolin	1 µM	105,10	0,14		119,33	0,17	*
	Leflunomide	10 µM	99,21	0,03		88,18	0,12	
		30 µM	95,18	0,07		89,21	0,14	
H_2O	Cobalt Chloride	200 µM	100,19	0,09		231,88	0,53	***
	LPS E. coli	2 µg/mL	98,87	0,09		102,24	0,07	
	Chloroquine	10 µM	99,86	0,09		73,37	0,06	***
Ethanol	Phenazine	50 µM	102,06	0,03		86,44	0,16	

C

Transfected cells/ non-transfected cells	% of cell viability	SD	*p*-value
Neutral Red Uptake Assay	81,50 %	0,17	*
Alamar Blue Assay	81,40 %	0,06	***

D

Solvent	Molecule	Concentration	% of cell viability	SD	*p*-value
Ethanol	Pyocyanin	50 µM	91,80	0,18	
		100 µM	88,57	0,15	

Table S2: Firefly luciferase inhibitory assay. Molecules were tested on purified firefly luciferase to evaluate their capacity to inhibit the enzyme. The Firefly luciferase inhibitor ß-naphthoflavone was used as a positive control of the assay. Six independent assays were performed in triplicate and statistical significances relative to the control (vehicle: DMSO or water) were examined: * *p*-value < 0,05; ** *p*-value < 0,01; *** *p*-value < 0,001.

Solvent	Molecule	Concentration	% of Firefly luciferase activity	SD	*p*-value
DMSO	ß-naphthoflavone	1 µM	13,07	0,01	***
	Hydroquinone	50 µM	100,99	0,04	
	3-methylcholanthrene	0,5 µM	97,49	0,06	
		5 µM	80,61	0,06	**
	Benzo(a)pyrene	0,5 µM	104,35	0,05	
	Fluoranthene	50 µM	99,98	0,04	
		100 µM	97,14	0 04	
	CH223191	1 µM	103,66	0,13	
	FICZ	50 nM	98,61	0,05	
		100 nM	92,48	0,06	
	SB202190	30 µM	101,50	0,08	
	Curcumin	100 µM	100,28	0,13	
	Clotrimazole	5 µM	100,91	0,07	
	Indole	250 µM	97,61	0,02	*
	Forskolin	1 µM	105,85	0,05	
	Leflunomide	10 µM	100,74	0,06	
		30 µM	97,16	0,03	
H_2O	Cobalt Chloride	200 µM	96,69	0,04	
	LPS E. coli	2 µg/mL	101,05	0,07	
	Chloroquine	10 µM	105,02	0,08	
Ethanol	Phenazine	50 µM	94,20	0,04	*
	Pyocyanin	50µM	81,67	0,05	***
		100 µM	77,01	0,12	***
Nonane	TCDD	10 nM	93,38	0,05	*

Table S3: Tested compounds with no significant effect. Molecules were tested on the screening model. Three independent experiments were performed in duplicate; fold induction is standardized to the vehicle, which is 1.

Molecule	Concentration	Fold induction	SD	*p*-value
2-aminoanthracène	10 µM	1,33	0,53	ns
3-phenylpropionic acid	10 µM	0,70	0,29	ns
	50 µM	0,87	0,38	ns
3-(4-hydroxyphenyl)propionic acid	10 µM	1,13	0,76	ns
	50 µM	1,18	0,66	ns
Indole-3-carboxaldehyde	10 µM	0,88	0,09	ns
	50 µM	1,03	0,11	ns
Indole-3-propionic acid	10 µM	0,78	0,18	ns
	50 µM	0,99	0,40	ns
Kynurenic acid	10 µM	1,01	0,14	ns
	50 µM	0,89	0,08	ns
Tryptamine	10 µM	0,98	0,16	ns
	50 µM	1,03	0,13	ns
Tryptophan	0,1 µM	0,90	0,07	ns
	1 µM	0,84	0,07	ns
	5 µM	1,00	0,27	Ns
Resveratrol	0,01 µM	1,00	0,03	ns
	0,1 µM	1,09	0,16	ns
	1 µM	1,05	0,44	ns
Quercetin	1 µM	0,98	0,15	ns
	10 µM	0,87	0,47	ns

Molecule	Concentration	Fold induction	SD	*p*-value
E. *Coli* pellet	25 µL	1,24	0,66	ns
Flagellin	100 ng/mL	0,90	0,40	ns
Dibutyryl-AMPc	2 µM	1,03	0,44	ns
3-3'diindolomethane	10 µM	1,26	0,31	ns
Biliverdin	16 µM	0,88	0,58	ns
	32 µM	0,88	0,38	ns
Colchicin	1 nM	1,19	0,34	ns
	1 µM	1,14	0,26	ns
Genistein	1 µM	1,09	0,34	ns
	10 µM	1,07	0,09	ns
Indigo	0,1 µM	0,92	0,37	ns
	1 µM	0,92	0,31	ns
ITE	1 µM	0,97	0,22	ns
	10 µM	1,01	0,22	ns
Lumichrome	1 µM	0,79	0,09	ns
	10 µM	1,10	0,18	ns
Pyridazine	5 mg/mL	0,96	0,20	ns
	50 mg/mL	0,85	0,31	ns
	500 mg/mL	0,92	0,26	ns
TCDD	10 nM	1,02	0,01	ns

Table S4: Primers used for the quantitative real-time polymerase chain reaction.

Genes	Primer sequences (5' - 3')
gcy-32	TCCCGTGTCAAAAACTCC
	AGCCATTCCAAGAGATTCC
gcy-33	CTTCCTTCAGCGACACCTTC
	GATCCTGCCATACTGGATCG
gcy-35	ATTACTCGAAGCGCAGTGGT
	TGCATCCAAATGTTCCTGTT
gcy-36	TGAAGCAGCAAAAAGGGTTT
	CAGTTCCAAGAGCCTCCTCA
cyp-13a10	TGTTCTCGGCTCAAGGATTT
	ATTCCATCGCACTGTCTTCC
cyp-13a1	CGCATTGGAGTTGTTGAGG
	TTTGAGAATCAGTTTGACCCATT
flp-8	ACCACCGAGAATGAGAAGGA
	CGTCACTGCGTTTTCCAA
ahr (human)	ACATCACCTACGCCAGTCGC
	TCTATGCCGCTTGGAAGGAT
arnt (human)	ACCAGCCACAGTCTGAATG
	TCTCCTTGAGCCCATACAC
cyp-1a1 (human)	GATCAAGGAGCACTACAAAACC
	TGGATATTAGCGTTCTCAT
